# Asking about violence and abuse among patients experiencing homelessness: a focus group study with healthcare professionals

**DOI:** 10.1186/s12913-024-10914-3

**Published:** 2024-04-26

**Authors:** Sophie Nadia Gaber, Ing-Britt Rydeman, Elisabet Mattsson, Åsa Kneck

**Affiliations:** 1 Department of Health Care Sciences, Marie Cederschiöld University, Stockholm, Sweden; 2 Department of Women’s and Children’s Health, Healthcare Sciences and e-Health, Uppsala University, Uppsala, Sweden

**Keywords:** Abuse, Delivery of health care, Health services accessibility, Health service research, Homeless persons, Qualitative methods, Violence

## Abstract

**Background:**

People experiencing homelessness are at increased risk of violence and abuse, however, there is insufficient knowledge about rates of inquiry or readiness of healthcare professionals to address violence and abuse among this population. This study aimed to explore healthcare professionals’ experiences and perceptions of asking about violence and abuse among patients experiencing homelessness.

**Methods:**

This study used a qualitative, interpretive, and exploratory design. We performed focus group discussions with healthcare professionals (*n* = 22) working at an integrative healthcare unit for people experiencing homelessness. Data were analysed using reflexive thematic analysis, following Braun and Clarke’s six-phase approach. Findings are reported according to the Consolidated Criteria for Reporting Qualitative Research (COREQ) checklist.

**Results:**

The overarching theme of the analysis is that addressing violence and abuse is at risk of “falling through the cracks”. The theme is supported by three sub-themes: *Hesitance to address violence and abuse*, *The complex dynamics of violence and abuse in homelessness*, and *Challenges in addressing violence and abuse amidst competing priorities and collaborative efforts.* The normalisation of violence and abuse within the context of homelessness perpetuates a “cycle” where the severity and urgency of addressing violence and abuse are overlooked or minimised, hindering effective interventions. Moreover, healthcare professionals themselves may inadvertently contribute to this normalisation. The hesitance expressed by healthcare professionals in addressing the issue further reinforces the prevailing belief that violence and abuse are inherent aspects of homelessness. This normalisation within the healthcare system adds another layer of complexity to addressing these issues effectively.

**Conclusions:**

The findings underscore the need for targeted interventions and coordinated efforts that not only address the immediate physical needs of people experiencing homelessness but also challenge and reshape the normalised perceptions surrounding violence and abuse. By prioritising awareness, education, and supportive interventions, we can begin to “break the cycle” and provide a safer environment where violence and abuse are not accepted or overlooked.

**Supplementary Information:**

The online version contains supplementary material available at 10.1186/s12913-024-10914-3.

## Background

Housing is a fundamental human right; however, every night, more than 700,000 people are sleeping rough in Europe, marking a 70% increase over the past 10 years [1]. Homelessness represents one of the most severe forms of poverty and deprivation and is associated with premature death [2] and trimorbidity (i.e., co-occurring physical and mental health issues and substance use disorders) [3, 4]. Previous studies have emphasised the fragile interconnection between violence, abuse, and homelessness [5], as violence and abuse can both be a cause and effect of homelessness [6]. People experiencing homelessness face heightened risks of victimisation, violence, and abuse compared to the general population [3, 7, 8], resulting in severe physical consequences (e.g., injuries, death), psychological implications (e.g., fear, trauma), and socioeconomic repercussions (e.g., loss of productivity) [3, 9, 10]. Violence and abuse significantly contribute to the disease burden among women of child-bearing age [9], with women experiencing homelessness being particularly vulnerable due to the high prevalence and detrimental impact of violence and abuse on their health and well-being [11]. Moreover, previous research has primarily focused on intimate partner violence, or domestic violence and abuse, within the home environment. This largely overlooks the high rates of violence and abuse among people experiencing homelessness (e.g., those sleeping rough or in shelters), especially among those living with substance use disorders [11] and a psychiatric diagnosis [8].

International and national policies call for the development of health and social care systems that can identify and tailor services to people experiencing violence and abuse [9, 12, 13]. This necessitates a multidisciplinary approach to address the complex and interconnected health and social care needs of people experiencing homelessness [8]. Literature increasingly focuses on the *readiness* of health and social care services to respond to patients exposed to violence and abuse, to identify gaps, and to understand individual and organisational *readiness* to implement interventions addressing violence and abuse [12]. The concept of *readiness* refers to facilitators that may motivate and empower people to make changes and improvements, including self-efficacy, motivations, and attitudes [9, 12, 14]. However, previous research has revealed low rates of inquiry about violence and abuse by healthcare professionals (approximately 10–30%) [9, 15], as well as low rates of disclosure by patients exposed to violence and abuse in healthcare settings [9, 15, 16]. Less is known about the rates of inquiry and *readiness* among healthcare professionals to address violence and abuse among people experiencing homelessness.

Providing training to healthcare professionals and equipping them with relevant information, screening tools, and skills may facilitate the identification of violence and abuse [9, 16]. However, there is a knowledge gap regarding systematic approaches to support, educate, and train healthcare professionals in responding to disclosures of violence and abuse, particularly among people experiencing homelessness. Therefore, to provide more tailored support that addresses the complex and intertwined needs of people experiencing homelessness, empirical research is necessary to explore how healthcare professionals perceive and comprehend violence and abuse among this population.

## Methods

### Aim

This study aimed to explore healthcare professionals’ experiences and perceptions of asking about violence and abuse among patients experiencing homelessness.

### Study design

This study used a qualitative, interpretive, and exploratory design. We analysed focus group discussions from healthcare professionals, collected as part of a broader workplace project that aimed to improve care for patients experiencing homelessness who are exposed to violence and abuse. The reporting of this study is conducted in accordance with the Consolidated Criteria for Reporting Qualitative Research (COREQ) checklist (Supplementary file 1) [17].

The present study focused on the part of the workplace project which involved implementing routines to inquire about violence and abuse. The purpose of implementing routines was to improve the detection of exposure to violence and abuse, and to strengthen connections to support services among patients experiencing homelessness. A working group of healthcare professionals was established, consisting of two managers (registered nurses) of the integrative healthcare unit, together with four staff members (one care coordinator, one psychologist, and two registered nurses) with a specific interest in developing care for patients exposed to violence and abuse. The focus of this present study is on healthcare professionals but the workplace included a multidiciplinary team (e.g., care coordinators/managers) which is more broadly referred to as staff here. Table 1 presents a timeline and overview of the project and its component activities (i.e., routines to address violence and abuse among people experiencing homelessness, such as establishing a working group and developing guidelines), in addition to the focus groups that formed the basis of the present study.

**Table 1:** Timeline for the project activities and data collection

Time point	Activities
November 2020	Project starts
January 2021	Working group formed
February 2021	Information and feedback with staff about the project (oral and written)
March 2021	Document templates developed
April 2021	Education sessions about violence and abuse offered to all staff Web-based training material on how to ask about violence and abuse developed and disseminated to all staff
May 2021	Written guidelines for asking and documenting about violence and abuse developed and implemented
September 2021	**Data collection I**
October 2021	Information and feedback with staff about the project (oral and written) Working group report back to the central management team Document templates developed Staff members attend externally arranged training on violence, abuse, post-traumatic stress disorder and trauma treatment
November 2021	Education sessions about violence and abuse offered to all staff Written material about the project developed and disseminated to patients (e.g., posters) Written procedures based on staff feedback revised
January 2022	Working group report back to the central management team Information and feedback with staff about the project (oral and written)
February 2022	Document templates developed Written procedures based on staff feedback revised
April 2022	**Data collection II** Project ends

Due to the COVID-19 pandemic, the project started later than intended and lasted one and a half years instead of the planned two years. The notes about discussions related to asking patients about violence and abuse, and these inspired topics that were explored among the healthcare professionals in the focus groups.

### Study setting

The study setting was an integrative healthcare unit, based on the integrative model of healthcare which combines physical, mental, social and spiritual dimensions of care delivered by a multidisciplinary team [18]. The integrative healthcare unit included a primary healthcare centre and a 24-hour ward with 45 employees. The unit is located in an urban area of Stockholm county, Sweden and provides services to registered citizens of the Stockholm healthcare region who are experiencing acute homelessness (based on categories one and two of the European Typology of Homelessness and Housing Exclusion [19]). The primary care centre is open on weekdays and offers drop-in and pre-booked appointments which are free of charge to the patient, in addition to an outreach care team providing mobile healthcare, and support with referrals to other health and social care services and civil society organisations (e.g., homelessness charities and voluntary organisations). The primary care centre provides psychiatric and somatic care, as well as treatment for substance use disorders and other services such as dental care and podiatry. The 24-hour ward primarily provides somatic care and has 12 beds. In 2020, the primary care centre had 1,503 registered patients and a total of 11,000 visits, and the 24-hour ward enrolled 120 patients resulting in 3,821 days of care. Approximately 70% of the patient population were male.

### Participants

The participants were recruited through convenience sampling. A written request was sent to all staff members (*n* = 45) who had daily contact with patients and those who were interested (*n* = 22, 49%) reported to the manager of the unit. Potential participants were provided with verbal and written information about the study, given time to discuss and ask questions about it, and provided written informed consent to participate. The manager formed the focus groups based on participants’ working schedules. Twenty-two people participated in the study, with eight people participating in both data collection I and II, while 14 people participated in only one of the data collections. The sample represented participants both from the primary care centre and the 24-hour ward. Table 2 presents an overview of the healthcare professionals who participated in the two focus groups for data collection I and the two focus groups for data collection II.

In data collection I, the sample consisted of 16 healthcare professionals (12 females and 4 males) and approximately 50% (*n* = 8) were aged between 51 and 60 years. In data collection II, the sample was comprised of 14 healthcare professionals (12 females and 2 males) and the majority were aged between 41 and 50 years and 51–60 years, 5 participants (35.7%) and 4 participants (28.6%) respectively.

**Table 2:** Overview of the characteristics of the participants in data collection I and II

Value	Data collection I (*n* = 16)	Data collection II (*n* = 14)
Female sex, n (%)	12 (75.0)	12 (85.7)
**Age group, n (%)**		
21–30		1 (7.1)
31–40	3 (18.8)	3 (21.4)
41–50	2 (12.5)	5 (35.7)
51–60	8 (50.0)	4 (28.6)
61–70	3 (18.8)	1 (7.1)
**Profession, n (%)**		
Assistant nurse, case manager, care coordinator, podiatrist	5 (31.3)	4 (28.6)
Physician, dentist	2 (12.5)	2 (14.3)
Nurse, occupational therapist, physiotherapist	7 (43.8)	6 (42.9)
Psychologist, counsellor	2 (12.5)	2 (14.3)

*Note* Data collection I (*n* = 8 + 8) and data collection II (*n* = 7 + 7). Eight people participated in both data collection I and II

### Data collection

This study was conducted in Stockholm, Sweden between September 2021 and April 2022. Data collection I was performed approximately halfway through the project and data collection II was performed at the end of the project (Table 1). All staff were offered general education and training in asking about violence and abuse before data collection I and then more focused education and training about violence, abuse, post-traumatic stress symptoms/disorder and trauma treatment prior to data collection II. The rationale for two data collection sessions was to gather richer data from the healthcare professionals following their education and training.

Two researchers facilitated the focus groups and provided questions and exercises to prompt discussions. However, the main focus was on the participants’ discussions and the generation of data through their exchange of experiences and opinions [20, 21]. The purpose of the focus groups was to gain insights about the *obstacles* and *possibilities* that the healthcare professionals perceived when asking about exposure to violence and abuse among their patients experiencing homelessness, in addition to reasons why patients may deny exposure to violence and abuse, and/or decline support when disclosing that they had previously or currently been exposed to violence and abuse. Open-ended questions and prompts were used in the focus groups, for example: *What are the obstacles and possibilities to asking people experiencing homelessness about exposure to violence and abuse as perceived by healthcare professionals? In general, what might cause patients to deny being victims of violence and abuse? Are there situations where you choose not to ask if the patient has been a victim of violence and abuse? Can you talk a bit about those situations?* The questions were developed for this study based on the literature [22]. The questions were not pilot tested. A list of the interview questions can be found in Supplementary file 2.

All focus groups were performed at the integrative healthcare unit with only the participants and focus group faciltators present, in order to minimise disturbances from colleagues and managers. The focus groups were audio recorded and lasted between 104 and 117 min (average 110 min). No field notes were taken. Data were processed and stored securely at the university to ensure confidentiality.

### Data analysis

The qualitative data were transcribed verbatim by a professional transcription service. The data obtained from the four focus groups were considered as a unified dataset and analysed inductively using reflexive thematic analysis, following Braun and Clarke’s six-phase approach [23, 24]. This method was used to identify, analyse, and report patterns of meaning across the data. The analysis process encompassed familiarisation with the dataset, coding, generating initial themes, developing, and reviewing themes, refining, defining and naming themes, and writing the analysis [23, 24]. The analysis involved an iterative process, comprising movement back and forth between the different phases. The data were manually organised using Microsoft Word®.

**Fig. 1:**
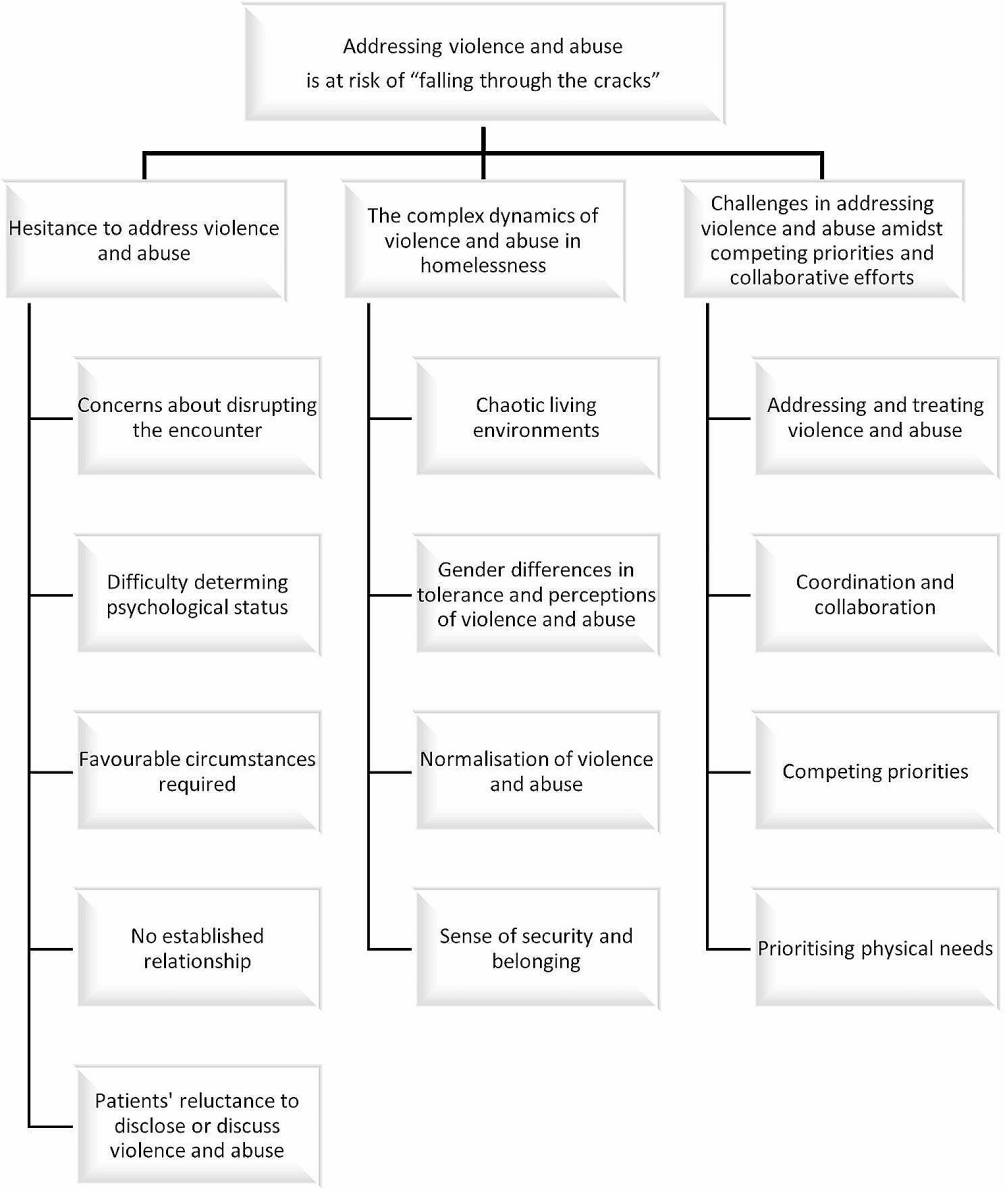
Thematic map presenting the overarching theme, sub-themes and codes

After familiarising ourselves with the dataset, concise labels were generated, referred to as codes. The labels captured and conveyed the significant features of the data, which were relevant for addressing the research aim. To gain a deeper understanding of the data, a semantic approach and a critical realist perspective were adopted in the coding process, considering both the explicit interpretations of the participants and the underlying social realities that influence those interpretations. One of the authors completed the coding of the transcripts, primarily engaging in discussions with one of the other authors. The next phase involved examining the codes and collated data to develop significant broader patterns of meaning within the dataset, which are referred to as potential themes. The development of themes was an iterative process, with themes being reorganised and refined throughout the ongoing phases of analysis. Themes were developed through analysing, combining, and comparing how codes related to one another. To gain a deeper understanding of the interconnected nature of the data, a thematic map was created, (Fig. 1).

This thematic map sought to capture the meanings embedded in the codes and facilitated the final reporting of the data. Through the process of creating the thematic map (Fig. 1), the complex relationships and interdependencies within the dataset were presented visually. The analysis process involved multiple reflexive meetings among the authors, to ensure that the identified codes, sub-themes, and overarching theme closely corresponded to the original data and accurately represented the entire dataset [23, 24]. Table 3 presents the steps in the data analysis.

**Table 3:** Examples from the analytical process

Quote	Code	Sub-theme	Theme
*Do you have dentures? Yes, I lost a prosthetic. Have you been subjected to violence? Well… it kind of creates a disharmony in the conversation, I think.*	Concerns about disrupting the encounter	Hesitance to address violence and abuse	Addressing violence and abuse is at risk of *falling through the cracks*
*[Healthcare professionals] who have had that perception, you know, that it doesn’t matter if she has been raped, she is a prostitute so it’s like… it won’t make a difference anyway…*	Normalisation of violence and abuse	The complex dynamics of violence and abuse in homelessness	
*And I wish that in Stockholm County, there was a unified approach within social services regarding the implementation of the Social Services Act, rather than each municipality making its own interpretation. Because that makes it impossible for healthcare to have a good collaboration with all municipalities since everyone works in such different ways. That would be a wish.*	Lack of coordination and cooperation	Challenges in addressing violence and abuse amidst competing priorities and collaborative efforts	

### Reflexivity

We, as a research team, consisted of four female authors, all holding doctoral degrees (PhDs). Three of us had backgrounds as registered nurses (I-BR, EM, ÅK), while one author specialised as an occupational therapist (SNG). Our employment roles varied, with one author serving as a postdoctoral researcher (SNG), one as a senior lecturer (I-BR), one as a professor (EM), and one as a lecturer (ÅK). The researcher who conducted the focus groups (ÅK) possessed prior experience in conducting interviews and facilitating focus group discussions, showcasing proficiency in qualitative research methods. Moreover, this team member had familiarity with some participants through previous interviews conducted with women experiencing homelessness at the healthcare centre. Additionally, two of the team members (SNG, EM) had prior experience conducting research with women experiencing homelessness. This experience included conducting interviews and involving women with lived experiences in the research process. Collectively, our team’s backgrounds and characteristics contributed to a nuanced understanding of the research topic and potentially influenced data collection and analysis.

To address reflexivity, we actively engaged in regular joint discussions, iteratively analysed the data, and recognised our significant role in knowledge production and interpretation. This reflexivity added depth and rigour to the analysis by considering the influence of our individual perspectives on data interpretation and theme construction. It ensured transparency and bolstered the credibility of the thematic analysis by providing a more nuanced understanding of how themes were derived and interpreted in relation to the data.

## Results

### Addressing violence and abuse is at risk of “falling through the cracks”

The overarching theme of the reflexive thematic analysis is that addressing violence and abuse is at risk of “falling through the cracks”. The theme is supported by three sub-themes: *Hesitance to address violence and abuse*, *The complex dynamics of violence and abuse in homelessness*, and *Challenges in addressing violence and abuse amidst competing priorities and collaborative efforts.*

The hesitance to address violence and abuse is characterised by healthcare professionals expressing concerns about disrupting patient encounters, evoking negative emotions, and patients’ unwillingness to discuss the topic. This hesitance increases the risk of violence and abuse going unnoticed or unaddressed. The complex dynamics of violence and abuse in homelessness are observed in people experiencing homelessness finding a sense of security and belonging within their community, despite the presence of violence and abuse. The normalisation of violence and abuse within the community and among healthcare professionals contributes to the risk of overlooking or minimising these issues. Challenges in addressing violence and abuse amidst urgent needs and collaborative efforts arise from the prioritisation of immediate concerns, such as medical issues, food, and stable housing, over addressing violence and abuse. The lack of coordination and cooperation among different organisations further hinders effective intervention. This leads to the risk of violence and abuse being overshadowed by competing priorities. When these sub-themes are combined, they highlight the overarching theme that addressing violence and abuse is at risk of “falling through the cracks”.

### Hesitance to address violence and abuse

Healthcare professionals expressed hesitance about addressing violence and abuse when there was no established relationship with the patient, as they recognised the importance of creating a sense of security and trust. They emphasised the need for favourable circumstances, such as sufficient time and privacy, as prerequisites for initiating discussions about violence and abuse. One healthcare professional described this hesitation, stating, *“Sometimes if they come in with a black eye, you don’t forget it, but if you realise it at the end, then I can feel uncomfortable just throwing out [questions about violence and abuse] if I know I’m going to say goodbye in a minute.”*

Additionally, healthcare professionals expressed concerns about routinely asking about violence and abuse, as it could potentially disrupt the encounter and evoke feelings of unease and anger in the patient. They also questioned the motivation and reasoning behind asking, given their experiences of patients not wanting to talk about violence and abuse, being unable to cope with it, or failing to follow through on discussing it. Most patients responded with a “no” when asked about violence and abuse, and those who answered “yes” rarely wanted to delve further into the topic.

Healthcare professionals described an informal approach of prioritising issues perceived as urgent by the patient and addressing other matters one-by-one to mitigate anxiety or negative reactions. However, the healthcare professionals reported that when violence and abuse were mentioned, they often encountered resistance and reluctance from the patient to discuss it further. Establishing a sense of security and trust with the patient was perceived as crucial for them to feel comfortable disclosing and sharing their experiences of violence and abuse. Consequently, multiple encounters and the establishment of a relationship with the patient facilitated this process.

Healthcare professionals felt uneasy asking about violence and abuse during brief encounters, such as drop-in receptions or outreach activities, where limited opportunities existed to establish a connection. The challenges of addressing violence and abuse were further magnified in the fast-paced clinical environment, where other issues needed attention and privacy. Therefore, healthcare professionals would inquire about violence and abuse only when they deemed that the timing and circumstances were appropriate. One healthcare professional highlighted this, stating, *“You focus on other things, and that comes more and more when the patient feels safe too…”*.

Even when patients were admitted to the 24-hour ward, it took time to establish trust. Healthcare professionals explained their approach of sitting by the patient’s bed without any specific agenda, but some also avoided eye contact to foster a sense of safety and security. Responsiveness and respect for the patient’s privacy were considered important. The healthcare professionals refrained from asking about violence and abuse when the patient exhibited agitation, aggression, or depression. Moreover, they avoided such questions out of concern that it might embarrass the patient or induce anxiety. One healthcare professional explained, *“I kind of downplay the question because I notice… or you kind of sense it to some extent, you’ve been talking for a little while and then you notice that this won’t end very well like this, somewhere I’ve made an assessment without knowing about it, that it won’t end very well [to ask about violence and abuse].”*

### The complex dynamics of violence and abuse in homelessness

The healthcare professionals explained that their patients often live in chaotic environments. Despite the pervasive presence of violence and abuse in their daily lives, the healthcare professionals observed that the living environment provides a sense of security, familiarity, and community for people experiencing homelessness. According to the healthcare professionals, this sense of belonging is often the only connection people experiencing homelessness have to a community, and they fear losing it. One healthcare professional shared an example:

I have encountered someone who faces threats and violence but chooses to stay because leaving means severing ties with the group, similar to leaving their family. They endure the relationship because they cannot leave and change their living situation… otherwise, they would lose everything they have.

The healthcare professionals discussed that even when people experiencing homelessness face physical abuse or exploitation from someone they are with, having someone by their side offers a sense of protection. Consequently, they may be reluctant to discuss violence and abuse with people outside their community, including healthcare professionals. The healthcare professionals noted that people experiencing homelessness may accept the “rules” within their community to maintain a sense of belonging.

Some healthcare professionals expressed that people experiencing homelessness rarely recognise the violence and abuse in their own lives, believing it to be a prerequisite for survival.

They don’t perceive it as violence because it’s the norm… hitting each other. Although they can acknowledge that it is violent, they haven’t questioned it at all. They may have numerous stories and episodes of being injured, but when asked directly, the answer is often ‘no’.

Furthermore, the healthcare professionals highlighted gender differences. They described a high tolerance for violence and abuse among some male patients, who viewed injuries as a “normal” part of their everyday lives.

Some patients are admitted due to violence, yet they downplay it, saying, ‘Yes, it was just a minor injury, not a severe assault.’ Living in an environment where such incidents are commonplace reduces the impact compared to our perception.

In response, the healthcare professionals aimed to encourage their patients to reflect on their experiences of violence and abuse, planting the seed during initial interactions and indicating their readiness to address it. They acknowledged the existence of violence and abuse and hoped that by creating awareness, future discussions could be fostered. However, some healthcare professionals also recognised that the normalisation of violence and abuse among people experiencing homelessness can impact their perceptions. Daily encounters with patients who are regularly exposed to violence and abuse may lead to a normalisation of these experiences among the healthcare professionals themselves.

It’s not only normalised in their group, but it’s also normalised within our group, I’m thinking of a patient who was robbed and lost his phone. At that point, we [healthcare professionals] were all discussing practical matters, like how to reach the patient to be able to admit him to the ward. But if it had been one of us, like if I had mentioned, ‘Oh, did you hear that X got robbed this morning on the way to work?’ then we would have been like, ‘Oh my God, what happened? How did it go?’ So, we tend to forget about it. It becomes somewhat normalised for all of us in different ways.

### Challenges in addressing violence and abuse amidst competing priorities and collaborative efforts

The healthcare professionals noted that people experiencing homelessness rarely seek support from the healthcare system specifically for their exposure to violence and abuse. Instead, healthcare encounters often focus on managing the consequences of violence and abuse, such as addressing injuries and practical issues.

The healthcare professionals explained that they prioritise addressing the immediate concerns that patients seek help for, especially if there are no obvious or acute injuries requiring immediate attention. Consequently, they feel that discussing violence and abuse may not be relevant if the patient requests assistance with other issues.

Patients often prioritise seeking help for other matters, and the order of priority ranges from medical tests and ECGs [electrocardiograms] to consultations with psychologists and counsellors. Consequently, the topic of violence and abuse tends to be set aside for later consideration.

Other urgent needs, such as insufficient food or stable housing, often take precedence for patients experiencing homelessness, pushing the issue of violence and abuse into the background. Recognising these challenges, healthcare professionals emphasised the importance of collaborating with other organisations to effectively support patients experiencing homelessness who are exposed to violence and abuse. They highlighted the responsibility of social services, for example, in arranging temporary accommodation, but also acknowledged the lack of coordination and cooperation among different organisations, which often depends on specific individuals or groups involved.

I feel a sense of frustration. It’s challenging to provide further help… Many at (the integrative healthcare unit) struggle to address issues of violence. As a healthcare professional, it’s frustrating not to be able to ensure a seamless process. The chain often breaks early, and I find that very difficult.

The healthcare professionals also emphasised the need for support from someone who can assist patients experiencing homelessness in organising and managing appointments and contacts, enabling positive changes in their living situations. The healthcare professionals described unique challenges when it comes to addressing and treating instances of violence and abuse. The diverse and complex needs that people experiencing homelessness face, often made it difficult to start treatment and planning.

This idea that social services, healthcare, that they have this ideal image of a crime victim and like… The ideal crime victim is easy to help because they don’t have multiple problems. But it’s not so easy to help someone who may also be violent themselves. I mean, then you can be disqualified, and if you’re mentally ill and, as we’ve talked about, if you also have a substance abuse problem, it clouds the vision.

Furthermore, the healthcare professionals felt that the added stress of homelessness made it even harder to support a person who has been exposed to violence and abuse as there is no escape from their dangerous and chaotic living conditions.

And then they need someone to help them with the planning itself. Sure, you can do that, but when you’re living on the streets, with no money and everything is unstable, you may have to start from scratch. Once they understand that, they seek help in making the right connections. But many individuals ask the same question: ‘How can I escape from my problems?’

## Discussion

The hesitance of healthcare professionals to address violence and abuse, the complex dynamics within the context of homelessness, where people may find security within their communities despite the presence of violence, and the normalisation of violence among healthcare professionals and the community, all contribute to the risk of violence and abuse “falling through the cracks”. Additionally, competing priorities and the lack of coordination of services further exacerbate the risk of these issues being overlooked or minimised, despite their significance.

Several studies have examined the obstacles to identifying and addressing violence and abuse [9, 12, 25]. In line with findings from other studies [26, 27], healthcare professionals identified insufficient time during encounters as an obstacle to addressing violence and abuse. In juxtaposition to previous research [9, 28], the healthcare professionals in our study did not discuss skills, emotional strain resulting from personal experiences of violence and abuse, or feeling overwhelmed by the emotional burden of the topic. It is not possible to know why the healthcare professionals did not discuss specific topics; however, we may infer that it in part relates to the normalisation of violence and abuse that emerged in our findings and this is discussed in more detail in the subsequent sections.

Healthcare professionals identified various obstacles to patients disclosing experiences of violence and abuse, including structural and organisational obstacles to accessing healthcare and social services. Literature has highlighted fragmented healthcare and social services located in disparate or hard-to-reach locations, bureaucratic processes, and limited operating hours as obstacles to the continuity of care in general, and to disclosing and addressing violence and abuse specifically [18, 22, 29]. Interpersonal barriers, such as shame, concerns about privacy, fear of judgment, and disbelief, have also been reported by patients [9].

Our study supports the importance of establishing trust and building a relationship as fundamental steps in addressing violence and abuse, as highlighted by healthcare professionals. Previous research indicates that people experiencing homelessness, particularly women, encounter mistrust, exclusion, and stigma in their healthcare encounters [22, 30]. Stigmatising attitudes towards women experiencing homelessness have been found to negatively impact the quality of care provided by nurses and nursing students [31]. In contrast, healthcare professionals who cultivate caring relationships can promote the health and well-being of their patients by creating a sense of safety, trust, and a feeling of being at home [31, 32], which can have transformative and potentially life-saving effects [22].

Our results shed light on the complex dynamics surrounding violence and abuse within the context of homelessness, revealing a concerning trend where such harmful behaviours are at risk of becoming normalised. The harsh realities faced by people experiencing homelessness can create an environment where violence and abuse are not only prevalent [8, 11] but also ingrained in their everyday lives. This normalisation of violence and abuse poses significant challenges not only for those directly affected but also for healthcare professionals who interact with this marginalised population.

Moral disengagement theory [33], inspired by Albert Bandura, can be utilised as a framework to understand the cognitive processes through which violence and abuse are normalised both by the healthcare professionals and the patients experiencing homelessness who are exposed to violence and abuse. The theory focuses on the cognitive mechanisms shaping behaviour and attitudes, that enable people to disengage from moral standards and justify their harmful actions [33]. According to this theory, people can disengage morally by employing various cognitive strategies, such as dehumanising the victim, minimising the harm caused, or blaming external factors. The healthcare professionals’ hesitance to address violence and abuse may reinforce the belief that violence and abuse are inherent aspects of homelessness, and thus they may inadvertently contribute to its normalisation.

Our findings indicate that healthcare professionals displayed a tendency to prioritise physical needs, considering them as more acute, potentially neglecting or undervaluing the psychological needs of patients exposed to violence and abuse. In a previous study including multiple experiments, people tended to minimise the presumed importance of others*’* psychological needs compared to their physical needs [34]. According to the authors [34], this represents a form of dehumanisation, treating people as having relatively “weaker mental capacities” due to the emphasis on bodily states over the presence of a thinking and feeling mind. Our findings did not indicate such overt stigmatisation; however, we identified a risk that prioritising immediate physical concerns within the healthcare context may inadvertently result in the oversight or minimisation of a person’s psychological well-being. Building on previous research, our findings highlight a significant issue in the perception and prioritisation of needs, emphasising the importance of recognising and addressing both the physical and psychological aspects of a person’s well-being to avoid perpetuating dehumanising attitudes and practices.

Addressing the normalisation of violence and abuse among people experiencing homelessness and healthcare professionals requires a multifaceted approach [8, 35]. First and foremost, healthcare professionals should be educated about the potential for normalisation and the detrimental effects it can have on the well-being of people experiencing homelessness. This can help them recognise and challenge the normalisation of violence and abuse in their interactions.

Additionally, interventions should focus on providing alternative models of behaviour that promote non-violence, empathy, and respect [35]. Offering trauma-informed care, by planning and providing services that recognise and respond to the effects of trauma [18], is crucial to address the underlying issues contributing to violence and abuse [8, 35]. Furthermore, implementing policies and procedures that ensure accountability for violence and abuse within the context of homelessness is necessary to “break the cycle” of normalisation.

### Strengths and limitations of the study

A strength of the setting was that the integrative healthcare unit comprised a diverse range of healthcare and social services in a single setting. Typically, people experiencing homelessness have multiple and complex health and social care needs which necessitates them to have to visit fragmented services in different locations [22]. However, the proximity of services in the integrative healthcare unit enabled the multidisciplinary team to work and communicate together and provided richer discussions from different perspectives in the focus groups.

Approximately 50% of the staff expressed an interest in participating in the focus group discussions. However, we do not know the reasons for those who did not want to participate, which is a limitation of our study. It is possible to speculate that those who expressed interest in participating had a specific interest in developing care for patients exposed to violence and abuse.

The distinction between the participants*’* own workplace project and the research project was explained. We acknowledge that some participants in the focus groups were involved in developing and implementing the project*’*s interventions, which might pose a risk that they did not feel comfortable speaking freely. Moreover, some of the participants were familiar with one of the authors who facilitated the focus groups, and her research on violence and abuse. However, we emphasised that everyone participated on equal terms and reassured them that there were no right or wrong answers. The primary focus was on facilitating open discussions and elaborating on addressing violence and abuse in clinical practice.

The combination of a semantic approach and a critical realist view in the analysis facilitated a reflexive analysis that took into account the interaction between the researchers, the data, and the social context in which the research was situated [23, 24]. However, although the data provides a comprehensive view of healthcare professionals’ experiences and perceptions of asking about violence and abuse among patients experiencing homelessness, we acknowledge that the understanding developed through this analysis can only ever be partial. Moreover, the transcripts were not returned to the participants for comment or correction, and the participants did not provide feedback on the findings. The findings will be presented to the participating integrative healthcare unit, and we will engage in further discussions based on the implications of our findings.

## Conclusions

Our findings shed light on the risk of violence and abuse being overlooked or minimised when addressing the needs of patients experiencing homelessness. One notable aspect revealed by the analysis is the normalisation of violence and abuse within the context of homelessness. This normalisation perpetuates a “cycle” where the severity and urgency of addressing violence and abuse are overlooked or minimised, hindering effective interventions. Moreover, healthcare professionals themselves may inadvertently contribute to this normalisation. The hesitance expressed by healthcare professionals in addressing the issue further reinforces the prevailing belief that violence and abuse are inherent aspects of homelessness. This normalisation within the healthcare system adds another layer of complexity to addressing these issues effectively. The findings underscore the need for targeted interventions and coordinated efforts that not only address the immediate physical needs of people experiencing homelessness but also challenge and reshape the normalised perceptions surrounding violence and abuse. By prioritising awareness, education, and supportive interventions, we can begin to “break the cycle” and provide a safer environment where violence and abuse are not accepted or overlooked.

### Electronic supplementary material

Below is the link to the electronic supplementary material.

Supplementary Material 1

Supplementary Material 2

## Data Availability

The datasets used and/or analysed during the current study are available from the corresponding author on reasonable request.

## Abbreviations

COREQ: Consolidated Criteria for Reporting Qualitative Research (COREQ) checklist
ECG: Electrocardiogram
Fig: Figure
