# Supplementary material for: Asking about violence and abuse among patients experiencing homelessness: a focus group study with healthcare professionals
Source: BMC Health Serv Res. 2024 Apr 26;24:531. doi: 10.1186/s12913-024-10914-3 (PMC11046839; doi:10.1186/s12913-024-10914-3)
Supplement: Supplementary file 2 — Supplementary Material 2 [file 12913_2024_10914_MOESM2_ESM.pdf]

Supplementary file 2

Focus Group Session 1

Question 1: We know from research that patients often deny experiencing violence and abuse in general, and it is particularly common among individuals experiencing homelessness. What do you think could be the reasons for this?

Question 2: What are the obstacles and possibilities to asking people experiencing homelessness about exposure to violence and abuse as perceived by healthcare professionals?

Question 3: In general, what might cause patients to deny being victims of violence and abuse?

Question 4: Are there situations where you choose not to ask if the patient has been a victim of violence and abuse? Can you talk a bit about those situations?

Follow-up Session – Focus Groups Session 2

Question 1: We know that among patients who report experiencing of violence and abuse, only a few agree to receive care and help related to this issue. What are your thoughts on the reasons why patients decline care or assistance in relation to being exposed to violence and abuse, both in general and specific reasons for individuals experiencing homelessness?

Question 2: If you could make unlimited suggestions, what are your reflections on how healthcare should be designed/organised to better serve patients experiencing homelessness who are also exposed to violence and abuse?
